# Chronic Insomnia Disorder across Europe: Expert Opinion on Challenges and Opportunities to Improve Care

**DOI:** 10.3390/healthcare11050716

**Published:** 2023-02-28

**Authors:** Jason Ellis, Luigi Ferini-Strambi, Diego García-Borreguero, Anna Heidbreder, David O’Regan, Liborio Parrino, Hugh Selsick, Thomas Penzel

**Affiliations:** 1 Department of Psychology, Northumbria University, Newcastle NE1 8ST, UK; 2 Department of General Psychology, Università Vita-Salute San Raffaele, 20132 Milan, Italy; 3 Sleep Research Institute, 28036 Madrid, Spain; 4 Department of Neurology, Innsbruck Medical University, 6020 Innsbruck, Austria; 5 Faculty of Life Sciences and Medicine, King’s College, London WC2R 2LS, UK; 6 Disorder Centre, Guy’s Hospital, London SE1 9RT, UK; 7 Department of Medicine and Surgery, University of Parma, Via Gramsci 14, 43126 Parma, Italy; 8 Insomnia and Behavioural Sleep Medicine Clinic, University College London Hospitals, London NW1 2PG, UK; 9 Interdisciplinary Centre of Sleep Medicine, Medicine Centre, Charité Universitätsmedizin, 10117 Berlin, Germany

**Keywords:** chronic insomnia, cognitive behavioural therapy, Europe, general practitioners, healthcare policy, healthcare providers, hypnotics, sleep, pharmacotherapy

## Abstract

One in ten adults in Europe have chronic insomnia, which is characterised by frequent and persistent difficulties initiating and/or maintaining sleep and daily functioning impairments. Regional differences in practices and access to healthcare services lead to variable clinical care across Europe. Typically, a patient with chronic insomnia (a) will usually present to a primary care physician; (b) will not be offered cognitive behavioural therapy for insomnia—the recommended first-line treatment; (c) will instead receive sleep hygiene recommendations and eventually pharmacotherapy to manage their long-term condition; and (d) will use medications such as GABA receptor agonists for longer than the approved duration. Available evidence suggests that patients in Europe have multiple unmet needs, and actions for clearer diagnosis of chronic insomnia and effective management of this condition are long overdue. In this article, we provide an update on the clinical management of chronic insomnia in Europe. Old and new treatments are summarised with information on indications, contraindications, precautions, warnings, and side effects. Challenges of treating chronic insomnia in European healthcare systems, considering patients’ perspectives and preferences are presented and discussed. Finally, suggestions are provided—with healthcare providers and healthcare policy makers in mind—for strategies to achieve the optimal clinical management.

## 1. Introduction

Approximately one in ten adults in Europe have chronic insomnia [1] and the overall prevalence is increasing [2]. Patients with chronic insomnia experience frequent and persistent difficulties initiating or maintaining sleep despite having adequate time and conditions to sleep [3,4]. They also experience significant daytime consequences affecting their functioning and wellbeing, such as fatigue, concentration issues, poor social and occupational functioning, cognitive impairment, and mood disturbances [3,4,5]. Furthermore, there is a reciprocal association between sleep and general health status [6,7,8]. Healthy sleep has recently been added as one of the pillars of cardiovascular health [9]. Patients with persistent insomnia are more likely to develop serious comorbidities such as cardiovascular diseases, diabetes, arterial hypertension, depression, anxiety, and cognitive functioning impairment [8,10,11,12]. Therefore, chronic insomnia poses a considerable burden on patients and healthcare systems [2,13].

Treatment approaches include non-pharmacological as well as pharmacological options, each with specific benefits and limitations [2]. Nevertheless, there are considerable unmet needs in the everyday clinical management of chronic insomnia which creates challenges for both treating physicians and patients. National and local differences in healthcare services lead to variable clinical care across Europe. In this article, we aim to identify these differences and compare and contrast the unmet needs with regard to disease management, physicians’ practices, and healthcare policies in western European countries. We further discuss future perspectives and key actions needed to achieve the optimal management of chronic insomnia in Europe.

## 2. Diagnosis

Despite the fact that sleep problems are quite common in the general population (>30%) and even more so among patients visiting primary care physicians (>50%), they are not routinely addressed in physician–patient consultations [14]. Thus, insomnia patients are often overlooked [15,16].

Recently, insomnia disorder has been redefined as a condition in its own right and should be managed and prioritised accordingly [3,4,17,18]. When sleep problems become a significant concern for a patient and impact their daily life, clinical intervention is required. Chronic insomnia shares similar symptomatology with acute insomnia, but chronic insomnia symptoms have lasted ≥3 months (Supplementary Table S1). However acute insomnia may become chronic for some individuals [3,19,20]. In a UK study, approximately 21% of those initially diagnosed with acute (short-term) insomnia went on to develop chronic insomnia [19]. A Canadian cohort study, conducted in 2007–2014, reported a 47% probability of persistent insomnia five years after an initial insomnia diagnosis [21]. A Swedish prospective longitudinal study, published in 2019, estimated a 43.2% prevalence of persistent insomnia one-year following the initial diagnosis [22].

**Table 1:** Medicines used in Europe as pharmacological treatments for chronic insomnia.

Treatment Type	Chronic Insomnia or Insomnia-Related Indication *	Contraindications ^†^	Warnings and Precautions	Side Effects ^‡^
**Dual orexin receptor antagonist**				
Daridorexant [23]	Chronic insomnia in adults Long-term use after reassessment (every 3 months)	Narcolepsy Concomitant use with strong CYP3A4 inhibitors	Risk of falls in the elderly, caution with driving and when operating heavy machinery Concomitant use of CNS-depressant substances or alcohol Worsening of depression and suicidal ideation, sleep paralysis, hallucinations, cataplexy-like symptoms Severe hepatic impairment	Headache, somnolence, dizziness, nausea, fatigue
**Benzodiazepines**				
Diazepam [24]	Insomnia severe, disabling, or causing extreme distress insomnia associated with anxiety Short-term (≤4 w ^§^)	Severe or acute pulmonary insufficiency Concomitant use of alcohol	Dependence ^ǁ^, withdrawal symptoms, rebound insomnia Amnesia Concomitant use of opioids	Drowsiness, reduced alertness, muscle weakness Paradoxical reactions Dependence symptoms
Flurazepam [25]	Insomnia severe, disabling, or causing extreme distress Short-term (≤4 w ^§^)	Long-term use Myasthenia gravis, severe pulmonary or hepatic insufficiency, respiratory depression, spinal and cerebellar ataxia Phobic or obsessional states, chronic psychosis Sleep apnoea Acute intoxication with alcohol, sedatives, hypnotics, analgesics, psychotropics Use in children	Dependence ^ǁ^, withdrawal symptoms Precipitates suicide when used as monotherapy in depression	Somnolence, reduced alertness, ataxia, dizziness, headache, dysgeusia Muscle weakness, risk of falls
Lormetazepam [26]	Insomnia severe, disabling, or causing extreme distress Short-term (≤4 w ^§^)	Long-term use Myasthenia gravis, severe respiratory and liver insufficiency, sleep apnoea Acute intoxication with alcohol, sedatives, hypnotics, analgesics, psychotropics Pregnancy and lactation	Dependence ^ǁ^, withdrawal symptoms, rebound insomnia Amnesia, psychiatric and paradoxical reactions	Angioedema, anxiety, decreased libido, bradyphrenia, tachycardia Headache, dizziness, sedation, somnolence, attention disorder, amnesia, vision or speech disorder, dysgeusia, vomiting, nausea, upper abdominal pain, constipation, dry mouth Pruritus, micturition disorder, asthenia, hyperhidrosis,
Nitrazepam [27]	Insomnia severe, disabling, or causing extreme distress Short-term (≤4 w ^§^)	Myasthenia gravis, acute pulmonary and severe hepatic insufficiency, respiratory depression, sleep apnoea Phobic or obsessional states, chronic psychosis Use in children	Dependence ^ǁ^, withdrawal symptoms Loss of hypnotic effect efficacy with prolonged use Precipitates suicide when used as monotherapy in depression Concomitant opioids, recall impairment	Numbed emotions, confusion, depression, muscle weakness, fatigue, ataxia Drowsiness, reduced alertness, headache, dizziness, diplopia
Temazepam [28]	Insomnia severe, disabling, or causing extreme distress Short-term (≤4 w ^§^)	Myasthenia gravis, neuromuscular respiratory weakness Acute pulmonary and severe hepatic insufficiency, severe respiratory depression, sleep apnoea Obsessional states Pregnancy or planning for pregnancy, use in children	Dependence ^ǁ^, withdrawal symptoms, rebound symptoms Loss of efficacy to the hypnotic effects with prolonged use Precipitates suicide when used as monotherapy in depression, anterograde amnesia, bereavement, psychiatric and paradoxical reactions Risk of falls, concomitant opioids	Drowsiness, light-headedness, numbed emotions, reduced alertness, confusion, ataxia, fatigue, dizziness, muscle weakness, double vision
Triazolam [29]	Insomnia severe, disabling, or causing extreme distress Short-term (≤4 w ^§^)	Myasthenia gravis, severe respiratory and hepatic insufficiency, sleep apnoea Co-administration with strong CYP 3A inhibitors Use in <18 years	Dependence ^ǁ^, withdrawal symptoms, rebound insomnia Loss of efficacy to the hypnotic effects with prolonged use Concomitant opioids or alcohol use Anterograde amnesia, psychiatric and paradoxical reactions	Somnolence, dizziness, ataxia, headache
**Benzodiazepine receptor agonists**				
Zolpidem [30]	Insomnia severe, disabling, or causing extreme distress Short-term (≤4 w ^§^)	Severe hepatic impairment, scute or severe respiratory depression Pregnancy and lactation, use in <18 years	Dependence ^ǁ^, rebound insomnia, loss of efficacy to the hypnotic effects with prolonged use Myasthenia gravis, sleep apnoea, long QT syndrome, somnambulism and associated behaviours, psychiatric and paradoxical reactions, suicidal ideation, depression Severe injuries, concomitant opioids	Somnolence, headache, dizziness, fatigue, diarrhoea, nausea, vomiting, abdominal pain, back pain, upper or lower respiratory tract infection Exacerbated insomnia, memory impairment, amnesia, anterograde amnesia
Zopiclone [31]	Insomnia severe, disabling, or causing extreme distress Short-term (≤4 w ^§^)	Myasthenia gravis, severe respiratory and hepatic insufficiency, severe sleep apnoea syndrome Lactose intolerance, concomitant alcohol intake Pregnancy and lactation, use in children	Dependence ^ǁ^, loss of efficacy to the hypnotic effects with prolonged use Suicidal ideation, depression, somnambulism and associated behaviours, psychiatric and paradoxical reactions, amnesia Concomitant opioids	Bitter taste
**Melatonin**				
Circadin [32]	Primary insomnia (poor quality sleep) in adults aged ≥55 years Short term (≤13 w)	Hepatic impairment	Renal impairment, autoimmune disease, lactose, glucose intolerance Pregnancy and lactation	None
**Phytotherapeutics**				
Valeriana [33] Hops + Valerian [34] Passiflora + valeriana [35] Melissa [36]	*Sleep aid:* Hops + valeriana Melissa Passiflora + valeriana *Temporary relief of sleep disturbances*: Passiflora + valeriana Valeriana	*Passiflora + valeriana:* Lactose and sucrose intolerance, use in <18 years	*Passiflora + valeriana:* pregnancy and lactation *Valeriana:* Lactose, sucrose, glucose intolerance, concomitant use of hypnotics, sedatives, excessive alcohol, pregnancy and lactation, use in <18 yoa	*Gastrointestinal*: Hops + Valerian Valeriana
**Antidepressants (off-label use)**				
Agomelatine [37,38]	None	Hepatic impairment Concomitant use of CYP1A2 inhibitors, use in <18 years and ≥75 years	Risk of hepatic injury—Surveillance of liver function is required in all patients Increased risk of suicidal behaviour	Insomnia, somnolence, dizziness, anxiety, back pain, fatigue, weight gain, nausea, diarrhoea, constipation, abdominal pain, vomiting, increased transaminases
Amitriptyline [39]	None	Recent myocardial infarction, any degree of heart block, cardiac rhythm disorder, artery insufficiency, severe liver disease Concomitant use of monoamine oxidase inhibitors (MAOIs), age <6 y	Hepatic impairment	Agitation, drowsiness, dysarthria, congested nose, micturition disorders, thirst, hyponatremia
Doxepin [40]	None	Mania, severe liver disease, glaucoma, urinary retention, lactation	Risk of increased suicidal thoughts, hepatic or renal impairment, epilepsy	Drowsiness, dry mouth, constipation, increased risk of bone fractures with concomitant use of SSRIs and TCAs
Mianserin [41]	None	Mania, severe liver disease, lactation, age <18 years	Risk of increased suicidal thoughts, haematological and hepatic reactions, cardiac effects, epilepsy, diabetes, hepatic, liver impairment, anticholinergic effects, hypomania, phaeochromocytoma, pregnancy	Drowsiness
Mirtazapine [42]	None	Concomitant MAOIs, age <18 years	Risk of increased suicidal thoughts, epilepsy and organic brains syndrome Bone marrow depression, jaundice, hepatic or renal impairment, cardiac diseases, low blood pressure, diabetes, hyponatraemia, serotonin syndrome, severe cutaneous adverse reactions Lactose intolerance, pregnancy and lactation	Insomnia, anxiety, confusion, abnormal dreams, fatigue, somnolence, sedation, headache, lethargy, dizziness, tremor, amnesia Orthostatic hypotension, dry mouth, nausea, diarrhoea, vomiting, constipation, arthralgia, myalgia, back pain, peripheral oedema, exanthema, weight gain
Trazodone [43]	None	Intoxication with alcohol or hypnotics, myocardia; infarction, age <18 years	Risk of increased suicidal thoughts, epilepsy, hepatic or renal impairment, cardiac disease, hyperthyroidism, micturition disorders, acute narrow angle glaucoma, jaundice Pregnancy and lactation	Suicidal ideation, suicidal behaviours
Trimipramine [44]	Depressive illness with sleep disturbance	Recent myocardial infarction, cardiac arrythmias, mania, severe liver disease, lactation	Risk of increased suicidal thoughts, diabetes, serotonin syndrome, QT interval prolongation, narrow angle glaucoma, liver function Pregnancy	Dry mouth, disturbance of accommodation, tachycardia, constipation, and hesitancy of micturition Drowsiness, sweating, postural hypotension, tremor and skin rashes. Interference with sexual function
**Antipsychotics (off-label use)**				
Levomepromazine Hydrochloride [45]	None	Patients with coma, risk of closed angle glaucoma, patients at risk of urinary retention, history of agranulocytosis. Dopaminergic agonists, concomitant (potentially) haemotoxic drugs, alcohol Pregnancy and lactation	Fever, sore throat, infection, jaundice, extreme temperatures Extrapyramidal side effects, tardive dyskinesia, epilepsy, coronary insufficiency, cardiovascular disorders, QT interval prolongation, hepatic, renal insufficiency Concomitant antipsychotics, elderly	Sudden death of cardiac origin Drowsiness
Olanzapine [46]	None	In patients at risk of narrow-angle glaucoma	Dementia and Parkinson’s disease, neuroleptic malignant syndrome, diabetes, lipid alterations, anticholinergic activity, hepatic function, neutropenia, QT interval, thromboembolism, epilepsy, tardive dyskinesia, postural hypertension Lactose intolerance, peanut or soya hypersensitivity Pregnancy and lactation, use in <18 yoa	Sudden cardiac death Somnolence, hypersomnia, lethargy, dizziness, akathisia, parkinsonism, dyskinesia, orthostatic hypotension, weight gain, dry mouth, constipation, rash, arthralgia, asthenia, fatigue, oedema, pyrexia Increased levels of prolactin, triglyceride, glucose, cholesterol, aminotransferases, uric acid. Decreased total bilirubin, eosinophilia, leukopenia, neutropenia Erectile dysfunction, decreased libido
Quetiapine [47]	None	Concomitant CYP450 inhibitors, HIV inhibitors, azole-antifungal agents, erythromycin, clarithromycin, nefazodone	Risk of increased suicidal thoughts Metabolic risk, extrapyramidal symptoms, tardive dyskinesia, sleep apnoea Alcohol or drug abuse, use in <18 yoa	Withdrawal symptoms, somnolence, dizziness, headache, extrapyramidal, dysarthria, abnormal dreams, suicidal ideation and behaviour. Dry mouth, constipation, dyspepsia, vomiting, dyspnoea Peripheral oedema, irritability, pyrexia, mild asthenia, blurred vision, tachycardia, palpitation, orthostatic hypotension Increased levels of prolactin, triglyceride, glucose, cholesterol, aminotransferases, uric acid, TSH. Decreased haemoglobin, and neutrophils, leukopenia, T4, T3
**Antihistamines**				
Diphenhydramine Hydrochloride [48]	Aid to the relief of temporary sleep disturbance	Stenosing peptic ulcer, pyloroduodenal obstruction	Myasthenia gravis, epilepsy, prostatic hypertrophy, urinary retention, narrow-angle glaucoma, asthma, bronchitis, chronic obstructive pulmonary disease, hepatic or renal impairment. Tolerance Concomitant alcohol, other antihistamines, lactose, glucose intolerance Pregnancy and lactation	Sedation, drowsiness, disturbance in attention, unsteadiness, dizziness, dry mouth. Fatigue
Doxylamine [49]	None	Concomitant MAOIs	Anticholinergic effects, concomitant CNS depressants or alcohol Asthma, increased intraocular pressure, narrow angle glaucoma, stenosing peptic ulcer, pyloroduodenal obstruction, bladder-neck obstruction	Somnolence, dizziness, dry mouth, fatigue
Hydroxyzine hydrochloride [50]	None	Prolonged QT interval, asthma, porphyria Pregnancy and lactation	Hepatic or renal impairment	Drowsiness, lassitude, dizziness, incoordination, paradoxical stimulation. Headache, psychomotor impairment and antimuscarinic effects.
Promethazine hydrochloride [51]	Insomnia in adults Short-term	CNS depression, concomitant MAOIs, age <2 y	May thicken or dry lung secretions and impair expectoration Severe coronary disease, bladder neck or pyloro-duodenal obstruction, Reye’s syndrome, ototoxicity Lactose, sucrose intolerance, strong sunlight Pregnancy and lactation, prolonged use (>7 days)	Drowsiness, dizziness, restlessness, headaches, nightmares, tiredness, disorientation Dry mouth, blurred vision, urinary retention Urticaria, rash, pruritus, anorexia, gastric irritation, palpitations, hypotension, arrhythmias, extrapyramidal effects, restless leg syndrome, muscle spasms, tic-like movements of the head and face.

Abbreviations: CNS, central nervous system; MAOI, monoamine oxidase inhibitor; SSRI, selective serotonin reuptake inhibitor; TCAs, tricyclic antidepressant; yoa, years of age; pts, patients; w, week(s). * Sorted by relevance: from chronic insomnia indication to off-label use in insomnia; ^†^ in addition to hypersensitivity to active ingredients or excipients; ^‡^ very common (≥1/10) or common (≥1/100 to <1/10); ^§^ including tapering off; ^ǁ^ physical and psychological dependence with high doses, long-term use, alcohol, or drug abuse history.

The diagnostic procedure involves the careful recording of a patient’s medical and sleep history that should include details on triggers, circadian factors, and sleep-awake pattern [2,52]. Sleep diaries and structured interviews using sleep-specific questionnaires can be used to assist in diagnosis [2,52], and evaluate the severity using psychometrics such as the insomnia severity index (ISI) [3]. A patient’s partner can be consulted to gather information on limb movements and breathing pauses during sleep. Other sleep disorders and conditions that may mimic insomnia symptoms should be excluded. Comorbidities, psychiatric and psychological history, and history of substance and medication use should also be assessed [2,52]. Actigraphy or polysomnography may also be considered [2] but they are not part of the routine exams used by general practitioners.

## 3. Burden

### 3.1. Epidemiology

The European Sleep Research Society and the European Insomnia Network review of epidemiological data published up until 2018 in selected European countries reported a 5.8–34.8% prevalence range, among adults (aged ≥18 years), for classification system-based insomnia diagnoses [53]. Germany, Hungary, Italy, the Netherlands, Spain, and the UK were at the lower end of insomnia prevalence (<10.0%) while Norway and Russia were at the higher end (≥20%) [53].

Several epidemiological studies published after 2018 reported varying levels of insomnia prevalence throughout Europe, however their different definitions of „insomnia patients” preclude conclusive estimations (Supplementary Table S2) [22,54,55,56,57,58,59,60,61,62,63,64,65,66,67,68,69,70,71,72,73]. Most studies based their insomnia case definitions on questionnaires. Three studies from Norway used the latest diagnostic criteria: two reported a 20% prevalence using International Classification of Sleep Disorders-3 (ICSD-3) criteria [63,67] and the third reported a 7.1% prevalence using proxy Diagnostic and Statistical Manual of Mental Disorders-5 (DSM-5) criteria [66]. During the COVID-19 pandemic, insomnia diagnoses increased compared with the pre-pandemic period (Supplementary Table S2) [57,58,59,62,72,74,75].

**Table 2:** Benefits, challenges, and unmet needs for different insomnia treatments.

Treatment Type	Evidence in Chronic Insomnia *	Treatment Benefits	Challenges and Unmet Needs
**CBT-I overall or by components [53]:** Sleep restriction Stimulus control Sleep hygiene education Relaxation therapy Cognitive reappraisal Cognitive control/worry time Paradoxical intention	Metanalyses evidence: Good overall sleep outcomes and follow-up stability assessments high-quality of evidence [2] Clinically significant outcomes in 49 RCT for sleep quality, sleep latency, remission, responder rates—Moderate quality of evidence [76] No RBC trial on each component’s efficacy [2] No evidence on daytime impairment efficacy [2]	Potentially long-lasting sleep improvements [76] Minimal side effects [76] Reduce exposure to medicines side effects [76] Favourable cost-effectiveness [76]	Rare availability [2,53] HCPs training [2] Some components are not suitable to specific populations–not all components match every patient [2,75,76] Lack of standardization [53] Each components’ evaluation of efficacy [2] and specific effects of each component [77] The observed effects cannot be attributed to specific therapy aspects due to the variability of components included in studies [77] Thorough investigation of side effects is lacking [2]
**Other psychotherapeutic approaches [2]:** Mindfulness-based therapy Hypnotherapy Light therapy Exercise	*Mindfulness:* Moderate to good overall sleep outcomes—poor quality evidence [2] Insufficient overall evidence [76] *Hypnotherapy*: good effect of sleep-onset latency—poor quality evidence [2] *Light therapy and exercise:* small to moderate effects—low quality evidence [2]	Absence of severe side effects [2] Exercise has positive psychological and general health impact [2] Light therapy and exercise may be used as adjuvant treatments [2]	Efficacy evaluations are missing [2] Efficacy in refractory to CBT-I patient groups Further research is needed to adequately appraise efficacy and explore use in CBT-I refractory patients [2]
**Dual orexin receptor antagonists**	Evidence on long-term use [23] Two placebo controlled RCTs reported significant improvement in sleep parameters (wake after sleep onset, latency to persistent sleep, total sleep time, daytime functioning) at month 1 and 3 [78,79]: reduction in sleep latency, improvement in sleep maintenance with similar effect across adult age groups (<65 y or ≥65 y) effect size remained stable at month 3 improved sleep onset and maintenance at month 3 and increased total sleep time (mean 6.5 h) by ~1 h self-reported improvements in concordance with objective measurements (polysomnography) All daytime functioning compartments were improved (in 50 mg dose group)	No evidence of dependence, abuse, or withdrawal symptoms [23] Older adults (≥65 y) are not at an increased risk of side effects morning residual effects [80] No need for dose adjustment in older adults (≥65 y) [80]	
**Benzodiazepines (BZ)**	Meta-analyses evidence: Short-term (≤4 weeks) efficacy [2,81,82]—high quality evidence [2] Sleep improvement [83,84,85] Increased risk for side effects [84,85] *Grading of evidence quality:* ESRS: high quality evidence for the short-term use of BZs as 2nd-line treatment [2] AASM grading of evidence: Moderate (temazepam) and high (triazolam) quality [86]	Triazolam and Flurazepam: Sleep onset and maintenance benefits [87] Temazepam: Sleep onset benefits *only* in 18–65 yoa [87] Sleep maintenance benefits only in ≥65 yoa [87] Useful in patients with anxiety and pain [77]	Risk of dependence, serious withdrawal symptoms, rebound effects [86,87]—Particularity with prolonged and non-monitored use [86] Daytime side effects [87] Potential for tolerance and dependence [2] Lack of long-term data [77] Not recommended in long-term use [2] Short to intermediate action [88] Associated with increased mortality [2] Naturalistic studies to assess long-term efficacy, safety and define the profile of patients predisposed to substance abuse [2]
**Benzodiazepine receptor agonists (BZRAs)**	*Meta-analyses evidence:* Short-term efficacy [2,81,82] Sleep improvement [83,84,85] Increased risk for side effects [84,85] *Grading of evidence quality:* ESRS: high quality evidence for the short-term use of BZRAs as 2nd-line treatment [2] AASM for zolpidem: very low quality [86]	Primarily useful for sleep-onset or maintenance insomnia [88]	Potential for tolerance and dependence [2] Possible unsuitable for patients with substance abuse tendency [77] Not recommended in long-term use [2] Associated with increased mortality [2] Naturalistic studies to assess long-term efficacy, safety and define the profile of patients predisposed to substance abuse [2] Sleep benefits and safety not adequately documented [88]
**Melatonin**	Small clinically relevant effect [2] *Grading of evidence quality:* ESRS: low—not recommended [2] AASM: very low—not recommended [86]	Favourable safety profile [77] Useful in patients with a substance-abuse tendency [77]	
**Phytotherapeutics**	ESRS evaluation: Limited evidence [2] No clinically relevant efficacy [2] Hops + Valerian: Insufficient evidence from CTs [34] Valerian [33]: Based on traditional use *Grading of evidence:* ESRS: low-quality [2] AASM (Valerian): low-quality [86]		
**Antidepressants**	No evidence in insomnia pts, with the exception of doxepin [77]	Without significant abuse potential [77] Useful in patients with comorbid mood disturbances, depression, anxiety or pain [77,88]	Risk of insomnia [38] Risk of liver injury [89]—risk minimisation measures [90] Risk to precipitate mania in bipolar depression patients [77]
**Antipsychotics**	No robust evidence in insomnia pts (only some in schizophrenia pts) [77] *Grading of evidence:* ESRS: low- to very-low-quality [2]	Useful in patients with comorbid psychosis or bipolar disorder [77] Useful in patients with a substance-abuse tendency [77]	Tardive dyskinesia although less common side effect is an important concern [77]
**Antihistamines**	Limited data in insomnia [77] *Grading of evidence:* ESRS: low- to very-low-quality [2] AASM for diphenhydramine: low quality [86]	Useful in patients with comorbid allergic symptoms or upper respiratory infections [77] Useful in patients with a substance-abuse tendency [77]	

Abbreviations: AASM, American Academy of Sleep Medicine; CBT-I, cognitive behavioural therapy for insomnia; ESRS, European Sleep Research Society; RCT, randomised controls trial; y, year(s); yoa, years of age. * Based on the grading of evidence evaluations of the European Sleep Research Society [2] and the American Academy of Sleep Medicine [76] (except for dual orexin receptor antagonists).

### 3.2. Disease Burden

Large prospective studies [91,92] and meta-analyses [11,93] have shown that insomnia increases the risk of cardiovascular diseases (CVD). In a Spanish study among >0.5 million workers, those with difficulties initiating sleep, short sleep duration, or unrestful sleep, had higher CVD risk scores than those without sleep problems (*p* < 0.001) [69]. In a population-based study among 1959 patients with hypertension in southern Germany, impaired sleep drastically increased the risk of CVD (HR: 1.76, 95% CI: 0.96–3.22) [94]. Furthermore, a large population-based prospective study conducted in Norway [92], showed that chronic insomnia significantly increased the risk of depression (OR: 2.38; 95% confidence interval [CI]: 1.91–2.98) and anxiety (OR: 2.08, 95% CI: 1.63–2.64). It was also associated with an increased risk of fibromyalgia (OR: 2.05, 95% CI: 1.51–2.79), rheumatoid arthritis (OR: 1.87, 95% CI: 1.29–2.52), osteoporosis (OR: 1.52, 95% CI: 1.14–2.01), asthma (OR: 1.47, 95% CI: 1.16–1.86), and myocardial infarction (OR: 1.46, 95% CI: 1.06–2.00) [92].

A large population-based cohort study conducted in the Netherlands with a 10–15-year follow-up, found that people who slept ≤6 h and had poor quality sleep had a higher risk for coronary heart disease (hazard ratio [HR]: 1.79; 95% CI: 1.24–2.58) and for CVD (HR: 1.63, 95% CI: 1.21–2.19) than those who had 7–8 h of sleep.

Lastly, the chronic insomnia-induced impairments in patients’ quality-of-life measured by the EuroQol 5 Dimension 5 Level (EQ-5D-5L) questionnaire were demonstrated in an analysis of the 2020 National Health and Wellness Survey (NHWS) in the UK, Germany, France, and Italy [95]. Of the overall 62.319 survey participants, those with severe insomnia (ISI 22–28) had a poorer self-reported health status (i.e., lower EQ-5D-5L scores) (mean: 0.56; standard error [SE]: 0.01) compared to those without clinically significant insomnia (ISI 0–7) (mean: 0.79; SE: 0.01; *p* < 0.001) [95].

### 3.3. Economic Burden

Insomnia costs an estimated 0.8 million disability-adjusted life years (DALYs) for 2004 in high-income countries and 3.6 million DALYs globally [96]. An analysis commissioned by the European Brain Council, which focused on all member states of the European Union (EU27), as well as Iceland, Norway, and Switzerland, reported a €35 billion total cost (€20 billion direct and €15 billion indirect) of sleep disorders in 2010 (i.e., including but not limited to chronic insomnia); direct healthcare costs including clinical management such as diagnosis, treatment, physician visits, and hospitalizations, and indirect costs including lost productivity due to missed working days or early retirement [97]. In 2010, insomnia was the ninth most expensive brain disorder in the EU27, accounting for 389,753 DALYs [98]. The weighted mean cost per patient was €790 (in purchasing power parities [PPP] 2010) for direct (€441) and indirect healthcare costs (€348), but costs varied substantially between countries (Supplementary Figure S1) [97].

On the basis of these findings, the European Sleep Foundation concluded that the direct and indirect costs associated with insomnia place a significant strain on Europe’s healthcare systems [2].

The analysis of the NHWS 2020 data suggested a high economic burden associated with chronic insomnia given that participants with severe insomnia (ISI 22–28) had substantially higher (*p* < 0.001) frequencies of insomnia-related absenteeism (23.4% vs. 5.6%), presenteeism (41.2% vs. 16.5%), reduced overall productivity (44.7% vs. 18.3%), and more frequent visits to general practitioners (GPs) (adjusted mean 8.55 vs. 5.74) compared with participants with no clinical insomnia (ISI 0–7) [99].

## 4. Treatment

Ideally, chronic insomnia treatment should aim primarily at improving sleep quality and duration as well as daytime functioning [88]. Sleep parameters commonly used in clinical trials to assess treatment efficacy in sleep research include sleep efficiency, sleep latency (SL), wake after sleep onset (WASO), and total sleep time (TST) [86,88].

Patients with chronic insomnia are a heterogeneous population, so treatment decisions should be based on the patient’s disease characteristics, medical history, and personal expectations and preferences. Current treatment options include first-line cognitive behavioural therapy for insomnia (CBT-I) and second-line various pharmacological treatments [2] (Table 1).

### 4.1. Non-Pharmacological Interventions

CBT-I is a multicomponent intervention encompassing cognitive and behavioural strategies [53]. It improves sleep by targeting dysfunctional or sleep-incompatible thoughts, feelings, and behaviours [53].

### 4.2. Pharmacological Interventions

Second-line pharmacological treatments include a variety of substances with diverse sleep benefits and safety profiles, ranging from older treatments such as benzodiazepines and benzodiazepine receptor agonists (BZRAs) [100] to melatonin and the recently approved orexin receptor antagonist (ORA) darodirexant [23] (Table 1). Several other medicinal or phytotherapeutic substances are also used off-label. Most of pharmacological interventions are indicated by the regulatory authorities as short-term treatments (Table 1).

### 4.3. Patient Journeys in Europe

Clinical care for patients with chronic insomnia varies across Europe due to regional disparities in practices and access to healthcare. In Spain, for example, public insurance is universal and the national healthcare system is funded by taxes at a rate of 71.1% in 2015, which is lower than the rates in Sweden (84%), the UK (80%), and Italy (75%) [101]. Patients in Spain are registered with a general practitioner (GP), who evaluates the need for continuing support and follow-up if the patient has a chronic condition [101]. Therefore, in Spain, patients seeking medical advice for chronic insomnia symptoms will first consult their GP, who may prescribe therapies, order diagnostic procedures, or arrange specialised care [101]. The clinical practice guidelines of the Ministry of Health for first-line therapy for insomnia advocate sleep hygiene as an adjuvant to medications [102]. These may be combined with some of the CBT-I components [102]. A recent survey of Spanish GPs, in the Majorca health area, reported that most of them recommend sleep hygiene (85.1%) and primarily prescribe benzodiazepines (33.4%) rather than CBT-I (14.2%) [102].

In the UK, the National Health Service (NHS) provides health services funded primarily through general taxation and secondarily from private insurance and out-of-pocket payments [103]. Patients can register with any GP practice in any location [103] and their GP becomes one of the three first points of contact for the healthcare system—the other two being emergency departments and walk-in dental clinics [103]. GPs will refer patients to specialised care if needed; patients with private healthcare will follow the same pathway [103]. NICE and the British Association for Psychopharmacology have issued recommendations for insomnia, with CBT-I as first-line therapy and hypnotics reserved for use as short-term adjuvants for patients aged >55 years with severe symptoms [104,105]. Despite the guidelines, however, UK patients receiving benzodiazepines are likely to prolong their use. A survey conducted in 2014–2015 in Bradford, UK, among GP practices reported that 35% of benzodiazepine users were taking them for at least 12 times longer than recommended [106].

In Italy, the health service provides coverage to all residents funded through national and regional taxation, supplemented by out-of-pocket-payments for diagnostic procedures costs, pharmaceuticals, specialist visits, and non-urgent hospital visits [107]. Specialist care is provided directly in public hospitals or by accredited private providers, and individual specialists may provide both public and private care [107]. Patients register with a GP and are free to select primary, secondary, and specialised care from public or accredited private health providers based on their willingness to pay and the waiting lists [107]. A referral is required to receive public or private services [107]. As per the joint recommendations of five Italian scientific societies, CBT-I should be the first-line therapy for chronic insomnia depending on availability; in reality, CBT-I is rarely available [108]. Usually, it could take long time before a GP refers patients to the specialists. Patients with severe insomnia and comorbidities are referred to specialists for the comorbid disorder. If the GP thinks that the patient needs polysomnography, he/she would refer the patient to a sleep centre. In a large epidemiological survey of 738 GPs across Italy published in 2004, 18% of their patients with chronic insomnia were taking medications, primarily lorazepam (23%) and zolpidem (15%) [109]. A similar recent survey (2017–2018) reported that half of patients aged >50 (55.3%) had insomnia symptoms, of whom 45.6% were treated mainly with benzodiazepines [110].

The healthcare system of Switzerland is characterised by multiple private insurance plans with large physician networks. In most cases, patients opt for a referral system, i.e., they need a GP referral to access specialist services [111]. Patients may directly access any healthcare provider they choose if they have a private health insurance that allow such direct access. A survey conducted in 2018 among Swiss GPs reported that in patients with chronic insomnia without comorbidities only 8% of GPs recommended first-line CBT-I; 87% recommended sleep hygiene, 65% phytopharmaceuticals, 49% antidepressants, and 18% benzodiazepine receptor agonists [112]. Knowledge about CBT-I was poor among the GPs (19% reporting knowing nothing about it and 46% very little) and the majority (78%) reported not having knowledge of a specialist who could provide CBT-I [112].

In Germany, provision of care is divided into sectors with distinct organisational structures and governance [113]. GPs are the usual first point of contact but are not the gatekeepers to healthcare services [113]. Commonly, all patients have statutory health insurance and can visit an accredited specialist directly. Patients with private health insurance can visit any specialist they choose (no need to be accredited) [113]. Only patients voluntarily enrolled in the “GP-centred model” (Hausarztzentrierte Versorgung) need to be referred by a GP to specialists. In general, patients can freely visit hospitals without the need to be referred by a GP [113]. A cross-sectional study, conducted in 2012–2014, among adults aged >50 years insured by the German health insurance AOK North-West reported that prescriptions for benzodiazepines or BZRAs were mainly given for insomnia problems [114]. Most patients considered their intake to be vital for them and unemployment was a significant factor associated with long-term use (OR: 2.9, 95% CI: 1.2–7.1) [114].

In France, universal care is publicly funded, primarily through the statutory health insurance covering the whole population [115]. Although many facilities and resources are available, these are unevenly dispersed around the country. Both the public and private sectors provide healthcare under a semi-gatekeeping system, with GPs holding a key role [115]. Mental health care is provided in health and social healthcare divisions [115]. In 2015, 5.6% of the French population took benzodiazepines for sleep problems [116]. A 2015 study among GP and pharmacy patients found that approximately 15% had been prescribed CBT-I but 69% of GP prescriptions involved benzodiazepines [117].

In Sweden, Finland, and Norway, GPs have a central role in patients’ pathways and the usual care is pharmacological prescriptions from GPs [53]. An analysis of the Norwegian Prescription Database [118] showed that 45% of BZRA users had been receiving BZRAs for at least 1 year and 17% for at least 4 years [118]. In older adults (aged ≥65 years), 25% were 4-year long-term recurrent users [118].

## 5. Unmet Needs

Available data suggests that, in Europe, individuals with chronic insomnia will likely follow a path through the healthcare system that mostly involves sleep hygiene and over-the-counter medications followed by long-term pharmacological treatment [14]. Insomnia symptoms usually persist in the long term [119], and patients may make an often difficult trade-off between the benefits from pharmacological treatments and their side effects, leaving them with a number of unmet needs.

### 5.1. Treatment Considerations

Patients in Europe seldom get access to CBT-I, because of the lack of access and reimbursement or because of individual preference, or even the lack of awareness by the healthcare professionals (HCPs) [75]. Evidently, in practice, the evidence-based recommendations for using CBT-I as the first-line treatment for chronic insomnia are not followed [2]. Digital (internet-delivered) CBT-I, which has shown reasonable efficacy and effectiveness in clinical studies, meta-analyses, and real-world evidence analyses, is an important step forward in making CBT-I more accessible [1]. Additionally, in a randomised-controlled study, a statistically significant superior effect was reported with face-to-face CBT-I as compared to online treatment (Cohen d = 0.9) [120].

Regarding second-line pharmacotherapy, clinicians are not adequately aided in making medication sequencing or combination decisions in the absence of clear sets of standards [88]. Therefore, medication choices and prescription habits are not optimal [121]. Many substances are used off-label and without a clear evidence of benefit in patients with chronic insomnia [77] (Table 2), while their safety profiles also make them suboptimal choices. Benzodiazepines and BZRAs are effective for a short duration of treatment (4 weeks) but limited data are available on their efficacy in the long-term [122]. A two-week regimen (with two weeks withdrawal period) rarely works in patients with chronic insomnia [123] but their long-term use puts patients at risk of dependence, withdrawal, and rebound symptoms (Table 1 and Table 2). They may also induce tolerance [124], and falls, which are particularly dangerous in older adults [125]. Lastly, there is a lack of evidence on their administration in younger patients [77] and in patients with cognitive impairment or substance abuse disorder [77]. Moreover, the analysis of the 2020 NHWS survey showed that despite receiving pharmacological treatment, patients continued experiencing frequent moderate-to-severe insomnia episodes, causing a substantial burden on their quality of life. Interestingly, in the cohort of patients with chronic insomnia, those who were receiving insomnia medications had a poorer self-reported health status (mean, 0.68; SE, 0.26) compared to those who were not receiving medications (mean, 0.72; SE, 0.24; *p* = 0.002) [95].

### 5.2. Treatment Recommendations and Non-Adherence

Adherence to CBT-I could not predict treatment outcome in a recent systematic review [126], but this was likely due to the high degree of heterogeneity in methods used to assess adherence and the variety of factors and measures assessed to predict treatment outcome. To fully understand how CBT-I adherence affects treatment outcomes, sleep research studies must employ a standardised method of measuring adherence and treatment outcomes [126].

Side effects are the main factor associated with patient nonadherence to HCP treatment recommendations [127]. The severity of insomnia and benzodiazepine withdrawal symptoms may drive patients to extend intake, leading to tolerance and dose escalation [127,128]. In an Italian study, some patients who unsuccessfully tried to abruptly discontinue benzodiazepines ended up taking them for over three years; 64% of all patients with insomnia in this study had been on benzodiazepines for >3 years [121]. In a study from the Netherlands, more severe insomnia symptoms were associated with a lack of compliance with benzodiazepine therapeutic regimens [128].

### 5.3. Treatment Choices and Expectations

Current treatments don’t meet all the goals of therapy, and defining efficacy in insomnia therapy is challenging [86]. Long-term treatments need to provide convincing evidence that effectiveness in terms of improved sleep efficiency, SL, TST, or WASO [86] is not at the expense of patient safety [14]. Therapeutic objectives should be discussed with patients and customised to their needs [88]. Setting expectations for treatments is very important for treatment success. For instance, in CBT-I, ensuring that the patient understands the reasons for the various techniques employed, especially sleep restriction whereby the individual restricts their sleep opportunity, i.e., time in bed to more closely match their current levels of sleep ability and the average actual sleep time, helps them anticipate and manage the challenges of undertaking CBT-I [129]. For BZDS and BZRAs, sedation needs to be explained as a side effect and not as an efficacy measure.

Several factors may influence therapy selection, such as the patient’s previous experience and preference as well as the presence of comorbidities and treatments’ safety profiles, contraindications, availability, and costs [88]. Ideally, a holistic approach to the treatment of chronic insomnia would bring together a multidisciplinary group of healthcare services—primary care, specialist care, behavioural therapists, physician assistants, and pharmacotherapy experts—and would consider patients’ individual needs, values, and preferences [14]. This approach could involve combinations of CBT-I with adjuvant pharmacotherapies [14]. A patient’s response to treatment should be reassessed regularly and treatment adjusted accordingly [88].

### 5.4. Physician-Related Factors

A qualitative study, published in 2019 and conducted in Germany, assessed HCPs’ (physicians, nurses, and pharmacists) views on the long-term use of benzodiazepine and BZRA [130]. According to the HCPs, such prescriptions were frequently issued within hospitals and renewed by community GPs [130]. Overcrowding in physician waiting rooms and limited time for patient-physician consultations prevented thorough examination of individual patient needs, impeding treatment decision-making [130]. It was also reported that physicians frequently lacked resources and knowledge about treating insomnia, especially in non-responders [130].

According to a qualitative study published recently (2019) on 2013–2014 interviews with sleep specialists and GPs on the medical management of insomnia, sleep care given in the UK was insufficient, mostly due to funding and organisational issues [131]. Access to sleep centres was mainly focused on conditions such as sleep apnoea or narcolepsy rather than insomnia [131]. This study also reported that there was a paucity of basic sleep medicine education in the UK [131]. As recorded in the same study, GPs might know more about the pharmacology of the drugs they prescribe for insomnia than the condition itself. This raises concerns about whether chronic insomnia is recognised as a serious condition [131]. HCPs receive insufficient sleep medicine training at both undergraduate and postgraduate levels [53,132].

### 5.5. Patients’ Perceptions and Preferences

Although patient preferences should be considered in treatment decisions [86], patients frequently do not know whom to discuss treatment-related issues with [130] or are not involved in treatment choices [133] (Supplementary Figure S2). Some patients have integrated sleep medications into their daily lives due to habit, addiction, or an inability to recognise dependence [130].

In a recent discrete choice experiment that included patients with chronic insomnia from Germany, patients’ choices for sleep medications were driven by the impact on daytime functioning (33.7%), and patients would accept an increase in abnormal thoughts and behaviours to improve daytime functioning or avoid withdrawal effects [134]. These data suggest that a patient-centred approach would favour daytime functioning [134]. Furthermore, a systematic review of qualitative research [135] reported that patients lacked awareness about treatment side effects. Providing patients with educational resources including up-to-date guidelines, detailed information on CBT-I, and pharmacotherapy would improve their understanding of their condition and help them to make informed decisions about insomnia management [135]. The educational material could be delivered to patients through websites or by GPs, nurses, or community pharmacists [135].

## 6. Challenges

Taking all this into account, we believe that most of the challenges in chronic insomnia management are primarily relevant to HCPs and healthcare authorities.

### 6.1. HCPs

Lack of standardised training and qualification of HCPs involved in the management of chronic insomnia leads to inconsistencies in treatment and risks harm to patients. Despite the availability of specific curricula, many HCPs are not fully equipped to implement diagnostic procedures and manage patients’ long-term needs [136]. As more medical disciplines become involved in this field, it is imperative to expand educational initiatives in undergraduate and postgraduate medical students and continuing medical education courses [16,136]. The recently updated “catalogue of knowledge and skills” [137] of the European Sleep Research Society could help guide such educational endeavours.

There is no consensus about when lifestyle and behavioural therapies should be considered to have failed and hence HCPs lack clear guidance on the management of treatment failure, treatment sequencing, combination treatment, or patient’s referral to sleep specialists.

Off-label long-term use of benzodiazepines and BZRAs in insomnia continues, despite the availability of European and country-level guidelines raising awareness about the risk of dependence and abuse.

Most European countries lack professional bodies for HCPs involved in managing chronic insomnia. National professional organisations might direct educational programmes and aid healthcare authorities in optimising the insomnia management network in their country. In that respect, much still needs to be done to increase the network of sleep experts who provide CBT-I training to GPs.

HCPs routine patients’ assessment should involve questions on sleep quality and insomnia problems, as well as the impact of those problems on daytime mood and functioning.

Even when GPs dedicate adequate attention to patients with insomnia and consider referring the patient for CBT-I, they lack information on the geographic distribution of accredited sleep centres/labs and CBT-I professionals in the different European areas.

The scientific societies of GPs and sleep specialists should collaborate to share operational strategies for the clinical management of insomnia patients, thereby improving the real-life care delivered to these patients.

The general population lacks education on the aspects of sleep problems and needs expert guidance on self-medication habits. Plain language summaries and a variety of visual aids, books, and audio resources [53] could help disseminate up-to-date evidence-based information to patients [14].

In parallel, there is a need to foster patient representation in decision-making through patient advocacy groups.

### 6.2. Healthcare Authorities

Chronic insomnia is not prioritised by healthcare planners, managers, and funders, rendering effective therapeutic management more difficult. As a result, patient pathways through the healthcare systems often result in suboptimal treatment approaches and outcomes.

Reliable high-quality data on the prevalence of insomnia are required at the European level. Eurostat’s European Health Surveys, which have been conducted in waves since 2006, could be used to obtain this type of information [138]. Future such surveys should include detailed sleep indicators, i.e., sleep quality and duration and daytime consequences of sleep.

At the national level, the diverse geographical distribution of sleep specialists and centres generates coverage gaps. Furthermore, access to specialised sites is neither planned nor prioritised. Such difficulties should be addressed at the organisational level, and provisions should be established to ensure that all patients have equal and unhindered access to specialised first-line therapies. With the move to hybrid working and the increase of online interventions, an opportunity has been presented to reduce geographical disparities in the provision of healthcare.

## 7. Conclusions and Future Directions

Chronic insomnia has a major, sometimes unnoticed, impact on patients, and healthcare systems. Inadequate management of chronic insomnia increases the overall burden of diseases in Europe and is the source of considerable healthcare costs. Despite this, chronic insomnia is still neglected as a major health concern in European healthcare systems. This must change if we are to provide the levels of treatment to which European residents are entitled. The current management approach is suboptimal, partly because existing medicines are used sub-optimally (and more than often off-label for pharmacological treatments) and therapeutic gaps exist that must be filled with novel approaches and medications. To adequately address these demands, sleep medicine should universally be standardised and recognised as a specialty. In that respect we suggest the following priorities for the way forward:

Scientific associations for sleep medicine should be established across European countries.

Insomnia management should be included as a separate medical topic at the undergraduate level and should be part of formal GP and relevant specialty training.

Reliable and accessible patient information sources should be developed.

Patient education and patient societies should be prioritised.

HCPs in each country should be trained on agreed-upon insomnia identification and management courses, including CBT-I, pharmacotherapy, and broader management strategies.

Access to digital CBT-I should be increased.

Pharmacotherapies could be used as adjunctive therapies to first-line CBT-I, or as an alternative when CBT-I is unsuccessful or not accessible. Further data on adjunctive therapy with CBT-I needs to be made available.

At the national level, a network of specialist sleep centres should be established. The centres should be included in proactive healthcare planning to effectively close CBT-I access gaps and collaborate with GPs as first line healthcare professionals to treat chronic insomnia.

Sleep research should investigate additional therapies with novel mechanisms of action and further characterise the long-term impacts of chronic insomnia and long-term effectiveness of treatments.

## Data Availability

Not applicable.

## Supplementary Materials

The following supporting information can be downloaded at: https://www.mdpi.com/article/10.3390/healthcare11050716/s1, Table S1: Diagnostic features of chronic and short-term insomnia in different disease classifications/coding systems. Table S2: Epidemiology data for insomnia in Europe published in 2018–2022. Figure S1: Per-patient costs of sleep disorders in Europe (€PPP adjusted per capita2010). Figure S2: Coordinated care and engagement with patient preferences in selected European countries—Data from the 2014, 2015, and 2016 Commonwealth Fund International Health Policy Surveys.

## Abbreviations

AASM	American Academy of Sleep Medicine
BZRA	benzodiazepine receptor agonist
CBT-I	cognitive behavioural therapy for insomnia
CI	confidence interval
CNS	central nervous system
DALYs	disability-adjusted life year
DSM	diagnostic and statistical manual of mental disorders
ESRS	European Sleep Research Society
GP	general practitioner
HCP	healthcare professionals
HR	hazard ratio
ICD	international classification of diseases
ICSD	international classification of sleep disorders
MAOI	monoamine oxidase inhibitor
NHS	National Health Service
OR	odds ratio
PPP	purchasing power parities
SL	sleep latency
SSRI	selective serotonin reuptake inhibitor
TCA	tricyclic antidepressant
TST	total sleep time
WASO	wakefulness after sleep onset
