# Supplementary material for: Chronic Insomnia Disorder across Europe: Expert Opinion on Challenges and Opportunities to Improve Care
Source: Healthcare (Basel). 2023 Feb 28;11(5):716. doi: 10.3390/healthcare11050716 (PMC10001099; doi:10.3390/healthcare11050716)
Supplement: Supplementary file 1 [file healthcare-11-00716-s001.zip › healthcare-2113257-supplementary.pdf]

Supplementary Material

Supplementary Table S1*. Diagnostic features of chronic and short-term insomnia in different disease classifications/coding systems*

Classification / coding system	Common symptoms for both short- and chronic insomnia	Short-term insomnia	Chronic insomnia
ICSD3 (2014) [3]	Difficulty to initiate sleep<br>Difficulty to maintain sleep<br>Daytime symptoms:<br>• Fatigue, malaise<br>• Sleepiness<br>• Mood, behavioural disturbance<br>• Difficulties in social functioning<br>• Impaired work performance<br>• Increased accidents<br>• Concerns about sleep difficulties<br>Adequate opportunities & conditions for sleep<br>Sleep difficulties are not due to other sleep disorder	Symptoms' duration: <3 months<br>Symptoms' frequency: <3 per week	Symptoms' duration: ≥3 months<br>Symptoms' frequency: ≥3 per week
DSM-5 (2013) [17]<br>DSM-5-TR (2022) [18]	• Sleep duration ≤30 min<br>• Sleep for 20–30 min, mid-night walking and difficulty to resume sleep<br>• Waking ≥30 min earlier than desired (total sleep duration <6.5 hrs)<br>• Adequate conditions & opportunities for sleep<br>• Sleep difficulties are not due to other sleep disorder<br>• The sleep is not adequate to revitalise the person<br>• Daytime fatigue, sleepiness, irritability<br>• Cognitive impairment during the day	Symptoms' duration: 1–3 months	Symptoms' duration: ≥3 months<br>Symptoms' frequency:<br>• ≥3 nights per week<br>• ≥2 episodes in a year*:*
ICD-11 (2022) [4]	Difficulty with sleep initiation, duration, consolidation, or quality<br>Adequate opportunities & conditions for sleep<br>Daytime impairment:<br>• Fatigue, general malaise	Code 7A01<br>Persistent symptoms<br>Symptoms' duration <3 months	Code 7A00<br>Persistent, frequent, recurrent<br>Symptoms' duration ≥3 months

	• Depressed mood or irritability<br>• Cognitive impairment<br>Sleep difficulties are not due to other sleep disorder or substance or medication use<br>General sleep dissatisfaction<br>Sleep difficulties are an independent focus of clinical attention		Symptoms' frequency: ≥2 per week<br>Recurrent: Episodes lasting weeks over several years

Abbreviations: DSM, diagnostic and statistical manual of mental disorders; ICD, international classification of diseases; ICSD, International Classification of Sleep Disorders.

Supplementary Table S2. *Epidemiology data for insomnia in Europe published in 2018–2022**

Country, author (year)	Study design	Study population	Study period	Insomnia prevalence†
Eight European countries; Morin et al. (2021) [54]	• Cross-sectional (web-based) survey<br>• Insomnia Severity Index (ISI)	• General population<br>• 7,238 adults	2020	*ISI≥15:*<br>*Austria:* 12.5%; *Finland:* 13.6%; *France:* 19.0%; *Italy:* 8.2%; *Norway:* 22.2%; *Poland:* 24.4%; *Sweden:* 16.0%; *UK:* 25.6%
Finland; Saltychev et al. (2021) [55]	• Longitudinal repeated cohort surveys<br>• Changes in sleep quality over time	• Public sector cohort<br>• 38,400 employees	2000, 2004, 2008, 2012, 2016	36% with sleep difficulties
France; Savall et al (2021) [56]	• Cross-sectional survey<br>• ISI, Pittsburgh sleep quality index, Epworth sleepiness scale	193 firefighters	2018–2019	18.8% moderate-to-severe insomnia symptoms
France; Kokou-Kpolou et al. (2020) [57]	• Cross-sectional (web-based) survey<br>• ISI	• General population<br>• 556 adults	2020	19.1% (ISI≥15)
Greece; Voitsidis et al (2020) [58]	• Cross-sectional (web-based) survey<br>• Athens Insomnia Scale (AIS)	• General population<br>• 2,427 adults	2020	37.6% (AIS≥6)
Italy; Riva et al (2022) [59]	• Cross-sectional (web-based) surveys<br>• 3-point scale on sleep difficulties or insomnia.	• General population<br>• 883 respondents aged >16 years	2020	Reports of 'moderate" + "severe" sleep disturbances:<br>Before COVID-19 pandemic: 35.7%<br>During COVID-19 pandemic: 48.1%–74.4%
Italy; Dell'Osso et al (2022) [60]	• ISI	• General population<br>• 20,720 adults	2020	• Difficulty initiating sleep (severe + very severe): 6.1%<br>• Difficulty maintaining sleep: 7.4%<br>• Early wake up: 7.2%<br>• Daytime consequences: 10.7%<br>• Worried about the sleep: 3.8%<br>• Evident sleeping problem: 3.1%
Italy; Varghese et al (2020) [61]	• Cross-sectional survey	• General population<br>• 3,120 respondents aged >15 years	2019	• 29.5% insufficient sleep duration<br>• 14.2% sleep dissatisfaction

	• Structured questionnaire, computer-assisted personal in-house interview			
Italy; Rossi et al (2020) [62]	• Cross-sectional (web-based) survey<br>• ISI	• General population<br>• 18,147 adults	2020	7.3% (ISI≥22)
Norway; Sivertsen et al. (2021) [63,64]	• Prospective, epidemiological cohort study<br>• General questionnaire-modified Bergen Insomnia Scale (BIS)	• Tromsø (north Norway) general population<br>• 21,083 adults	2015–2016	*ICSD-3 insomnia case definition:*<br>Overall insomnia (ICDS-3): 20.0% insomnia (95% CI, 19.4–20.6)<br>Women: 24.8% (95% CI, 23.9–25.6)<br>Men: 14.9% (95% CI, 14.2–15.6)
Norway; Fetveit et al. (2019) [65]	• Prospective, epidemiological cohort study<br>• Self-reported frequency of sleeplessness	• Tromsø general population<br>• 12,982 adults	2007–2008	12.6% (sleeplessness >1 night/week):
Norway; Uhlig eт al. (2014) [66]	• Cross-sectional survey<br>• Sleep difficulties questionnaire	• Nord-Trøndelag county (central region) general population<br>• 40,535 adults	2006–2008	*Proxy DSM-V insomnia case definition:*<br>Overall: 7.1% (95% CI, 6.9–7.4)<br>Women: 8.6% (95% CI, 8.3–9.0)<br>Men: 5.5% (95% CI, 5.1–7.8)
Norway; Bjorvatn et al. (2018) [67]	• Cross-sectional (telephone)-survey<br>• modified BIS	• General population<br>• 1,001 adults	Not reported	*ICSD-3/DSM-5*<br>Overall: 20.0%<br>Female: 23.7%<br>Male: 16.3%
Spain; Torrens et al. (2019) [68]	• Cross-sectional (telephone)-survey<br>• ISI	• Majorca<br>• 467 adults	2010–2011	Clinical insomnia: 6.9% (ISI≥15)
Spain; Valenzuela et al. (2022) [69]	• Cross-sectional survey<br>• Questionnaire on sleep duration, difficulties fallings asleep, feeling of rest after sleep	• Workers with occupational risk prevention insurance<br>• 521,364 adults	TBD	33.0%
Sweden; Jansson-Fröjmark et al. (2019) [22]	• Longitudinal prospective, population-based, with 1-year follow-up<br>• Questionnaire on sleep hygiene, sleep, daytime symptoms, and daytime functioning	• General population<br>• 1638 adults	Not reported	8.1%

Spain; Zhang et al (2022) [70]	• Meta-analysis<br>• Studies published 2020–2021	• 3 studies / 5 samples / 745 participants<br>• ISI, PSQI	2020–2021	57.0% (95% CI; 48–66)
Sweden; Titova et al. (2022) [71]	• Cross sectional study<br>• Questionnaire on sleep duration, early awaking's, difficulties maintaining sleep, overall disturbed sleep, feeling of rest after sleep	• General population in Uppsala (east-central region) and Malmö (southwest region)<br>• 19,254 adults	Not reported	• 7.7% sleep initiation difficulties<br>• 9.0% sleep maintenance difficulties<br>• 11.0% early awakening<br>• 12.8% disturbed sleep<br>• 14.2% not rested after sleep
Sweden; McCracken et al. (2020) [72]	• Cross sectional, (web-based) survey<br>• ISI	• General population<br>• 1,212 adults	2020	• Moderate insomnia 14.7% (ISI 15–21)<br>• Severe insomnia: 3.1% (ISI 22–28)
Switzerland; Maire et al. (2020) [73]	• Cross-sectional	• Patients visiting GPs<br>• 2,432 adults	2018	• 36.0% with insomnia symptoms; 11.0% chronic

Abbreviations: AIS, Athens Insomnia Scale; BIS, Bergen Insomnia Scale; CI, confidence interval; GP, general practitioner; ISI, Insomnia Severity Index

[*]PubMed was searched for publications after 2017 containing epidemiological data on insomnia in European countries. Titles were screened on the basis of relevance to European insomnia data. The literature search was neither systematic nor exhaustive; [†]Insomnia prevalence as reported in corresponding publications

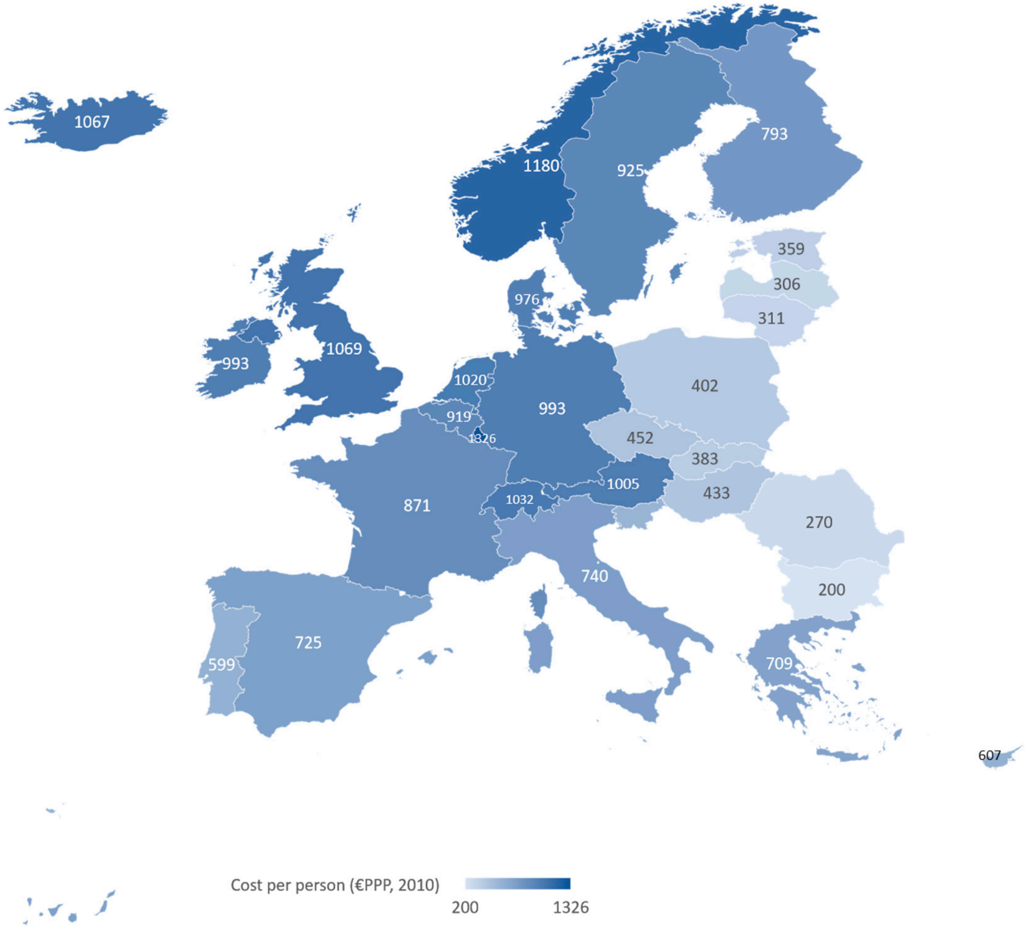

Supplementary Figure S1. *Per-patient costs of sleep disorders in Europe (€PPP adjusted per capita2010*).*

Source: Adapted from Gustavsson et al. (2011) [97]

Data are weighted mean for all ages from all countries (average for EU27, weighted by the total consumption in each country)

Abbreviations: PPP, purchasing power parities

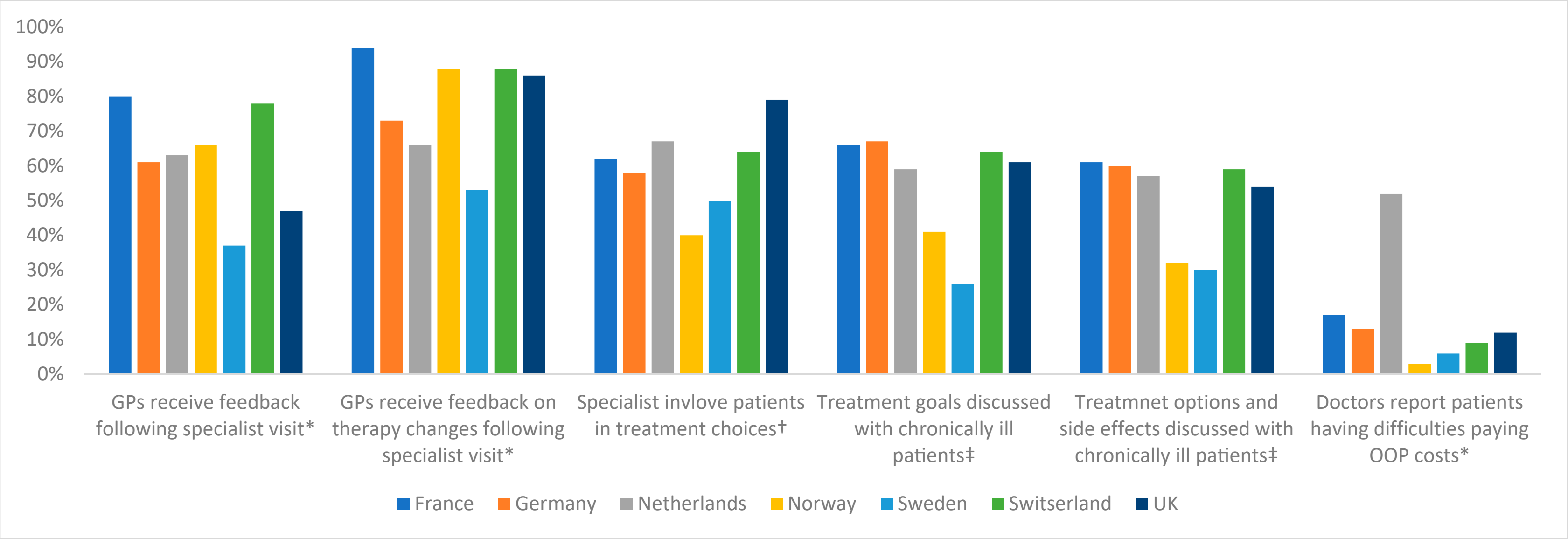

Supplementary Figure S2. *Coordinated care and engagement with patient preferences in selected European countries – Data from the 2014, 2015, and 2016 Commonwealth Fund International Health Policy Surveys.*

Source: Schneider et al. (2017) [133]

Abbreviations: GP, general practitioner; OOP, out-of-pocket

*responses received from GPs in the 2015 survey; †responses received from the general population in 2014 surveys; ‡responses received from the general population in the 2016 surveys
